# Gamma synuclein is a novel nicotine responsive protein in oral cancer malignancy

**DOI:** 10.1186/s12935-020-01401-w

**Published:** 2020-07-10

**Authors:** Chia-Chen Hsu, Yu-Fu Su, Kuo-Yang Tsai, Feng-Chih Kuo, Chi-Fu Chiang, Chu-Yen Chien, Ying-Chen Chen, Chien-Hsing Lee, Yu-Chiao Wu, Kun Wang, Shyun-Yeu Liu, Yi-Shing Shieh

**Affiliations:** 1 Graduate Institute of Medical Sciences, National Defense Medical Center, Taipei, 114 Taiwan; 2 Department of Radiation Oncology, Tri-Service General Hospital, National Defense Medical Center, Taipei, 114 Taiwan; 3 Department of Oral and Maxillofacial Surgery, Changhua Christian Hospital, Changhua, 500 Taiwan; 4 College of Nursing and Health Science, Da-Yeh University, Changhua, 515 Taiwan; 5 Division of Endocrinology and Metabolism, Department of Internal Medicine, Tri-Service General Hospital, National Defense Medical Center, Taipei, 114 Taiwan; 6 Department of Dentistry, Tri-Service General Hospital, National Defense Medical Center, No.161, Sec.6, Min-Chuan East Rd., Nei-Hu, Taipei, 114 Taiwan; 7 Molecular and Cell Biology, Taiwan International Graduate Program, Academia Sinica and Graduate Institute of Life Science, National Defense Medical Center, Taipei, 114 Taiwan; 8 Department of Internal Medicine, Cardinal Tien Hospital and School of Medicine, College of Medicine, Fu Jen Catholic University, New Taipei City, Taiwan; 9 Department of Oral and Maxillofacial Surgery, Chi Mei Medical Center, Tainan, 710 Taiwan; 10 Department and Graduate Institute of Biochemistry, National Defense Medical Center, Taipei, 114 Taiwan

**Keywords:** Gamma synuclein, Nicotine acetylcholine receptor, Oral cancer, Signal transduction

## Abstract

**Background:**

The mechanisms of neuronal protein γ-synuclein (SNCG) in the malignancy of oral squamous cell carcinoma (OSCC) are not clear. This study tested the hypothesis that SNCG is involved in nicotine-induced malignant behaviors of OSCC. The effect of nicotine on SNCG expression and epithelial-to-mesenchymal transition (EMT) markers were examined.

**Methods:**

Short hairpin RNA (shRNA) and an antagonist specific for α7-nicotine acetylcholine receptors (α7-nAChRs) were used to examine the role of α7-nAChRs in mediating the effects of nicotine. Knockdown of SNCG in nicotine-treated cells was performed to investigate the role of SNCG in cancer malignancy. The in vivo effect of nicotine was examined using a nude mouse xenotransplantation model.

**Results:**

Nicotine increased SNCG expression in a time- and dose-dependent manner. Nicotine treatment also increased E-cadherin and ZO-1 and decreased fibronectin and vimentin expression. After specific knockdown of α7-nAChRs and inhibition of the PI3/AKT signal, the effect of nicotine on SNCG expression was attenuated. Silencing of SNCG abolished nicotine-induced invasion and migration of OSCC cells. The xenotransplantation model revealed that nicotine augmented tumor growth and SNCG expression.

**Conclusion:**

Nicotine upregulated SNCG expression by activating the α7-nAChRs/PI3/AKT signaling that are participated in nicotine-induced oral cancer malignancy.

## Background

Oral cancer is the sixth most common type of cancer worldwide and the most common cause of head and neck tumors. Each year more than 500,000 patients are newly diagnosed with oral cancer [1], and more than 90% of these patients have oral squamous cell carcinoma (OSCC) [1, 2]. The overall 5-year survival rate for OSCC is less than 50% [3]. In the past 20 years, although advancements have been made in diagnosis and treatment, the mortality rate for oral cancer has not declined [2]. Investigating molecules that mediate OSCC progression could help enable early diagnosis and effective treatment.

Cigarette smoking is a risk factor for oral squamous cell carcinogenesis and progression [4–6], and nicotine is the major component of tobacco in cigarettes [7, 8]. Previous studies have indicated that nicotine promotes cancer progression in multiple types of cancer [4]. Nicotine exerts pathophysiological effects by binding to nicotine acetylcholine receptors (nAChRs). It has been reported that there are a high prevalence of nAChRs in the central nervous system (CNS) [9], and nAChRs are also observed in various nonneuronal cells, including cancer cells [4]. In the CNS, binding of nicotine to specific nAChRs leads to distinct electrophysiological and pharmacological properties. For example, α4β2-containing nAChRs have the highest nicotine-binding affinity in neurons [10]. After stimulation of α4β2 nAChRs, dopamine is released in the brain reward pathway, resulting in a smoking addiction. In cancer cells, binding of nicotine to nAChRs stimulates intracellular signaling pathways in a tissue-specific manner, activates downstream mitogenic pathways, and upregulates the expression of growth factors [11].

Synucleins are a family of homologous proteins consisting of three known members: α-synuclein (SNCA), β-synuclein (SNCB), and γ-synuclein (SNCG) [12]. Synucleins are abundantly expressed in the brain, especially in the presynaptic terminals of neurons [13]. Although the precise function of these proteins remains unknown, SNCA has been implicated in the pathogenesis of Parkinson’s disease (PD), Alzheimer’s disease and multiple system atrophy. SNCG expression is normally restricted to the brain and peripheral neuronal tissues [14], and its aberrant expression in tissues other than those of the neuronal system is highly associated with human malignancy. Previous studies have reported that nicotine acting on brain nAChRs may affect SNCA aggregation, resulting in neuroprotection [15–17]. Recently, we reported that SNCG is abnormally expressed in OSCC and that its expression is strongly correlated with disease progression [2]. However, at present, the molecular and cellular mechanisms underlying cancer-associated dysregulation of SNCG and whether SNCG is involved in the nicotine-induced malignant behavior of oral cancer remains unknown.

Therefore, the present study investigated the potential involvement of SNCG in mediating nicotine-induced oral cancer malignancy.

## Materials and methods

### Cell culture

Oral squamous cell carcinoma cell lines (OEC-M1 and YD8) were kindly provided by Professor Yook (Namseoul University, Korea) and Professor Meng (National Defense Medical Center, Taiwan). Cells were cultured in an RPMI1640 medium with 10% fetal bovine serum (FBS, Thermo Fisher Scientific, Waltham, MA, USA) before being incubated at 37 °C in a 5% CO_2_ atmosphere incubator. All oral cancer cell lines were confirmed to be free of mycoplasma.

### Chemicals

Nicotine was purchased from Sigma-Aldrich (St. Louis, MO, USA), the α7-nAChR antagonist methyllycaconitine (MLA) was purchased from Tocris Bioscience (Bristol, England, UK), the P-AKT inhibitor Ly294002 was purchased from Selleck chemicals (Houston, TX, USA). All other chemicals were obtained from Sigma.

### RNA extraction, polymerase chain reaction, and quantitative real-time polymerase chain reaction

mRNA was extracted from the cells with TRIzol reagent (Invitrogen, Carlsbad, CA, USA) in accordance with the protocol provided. Total RNA was reverse transcribed into cDNA using an oligo (dT) 12–18 primer to preserve the relative mRNA profile and produce a template suitable for a polymerase chain reaction (PCR). Quantitative Real-time PCR (QPCR) was performed using the SYBR-Green system in compliance with the protocol provided by Bioline (London, England, UK). Primer sequences were as follows: GAPDH (PCR), forward: GGT GAA GGT CGG AGT CAA CGG A; reverse: GAG GGA TCT CGC TCC TGG AAG A. GAPDH (QPCR), forward: CCA CAT CGC TCA GAC ACC AT; reverse: TGA CCA GGC GCC CAA TA. α7-nAChR, forward: GCT GGT CAA GAA CTA CAA TCC C; reverse: CTC ATC CAC GTC CAT GAT CTG. SNCG, forward: CAA GAA GGG CTT CTC CAT CGC CAA GG; reverse: CCT CTT TCT CTT TGG ATG CCA CAC CC. α3-nAChR, forward: CCA TGT CTC AGC TGG TG; reverse: GTC CTT GAG GTT CAT GGA. α5-nAChR, forward: TCA TGT AGA CAG GTA CTT C; reverse: ATT TGC CCA TTT ATA AAT AA.

### Protein extraction and Western blot analysis

Cell lysate combinations of RIPA with PIC2 and PPI were used to form the lysis buffer, and a reaction time of 30 min in an ice bottle was allocated. The protein concentration in each cell lysate was then measured using a commercial BCA kit (Thermo Fisher Scientific, Waltham, MA, USA). In the Western blot analysis, an SDS-polyacrylamide gel electrophoresis system was used. An anti-GAPDH antibody (Cell Signaling, Danvers, MA, USA), anti-fibronectin antibody (Abcam, Cambridge, England, UK), anti-vimentin antibody (cell signaling), anti-E-cadherin antibody (BD Biosciences, Franklin Lakes, NJ, USA), anti-ZO-1 antibody (BD Biosciences), anti-PCNA antibody (Cell Signaling), anti-protein kinase B (AKT) antibody (Cell Signaling), anti-phospho-AKT (P-AKT) antibody (Cell Signaling), anti-α7-nAChR antibody (Abcam), and anti-SNCG antibody (Cell Signaling) were used for probing.

### Wound-healing assay

Cells were seeded in a 6-well plate, and a 1000 µL pipette tip was used to form a wound after the cells had attached. The wound was washed three times with phosphate buffer to remove debris and then treated with 1 µM nicotine, 10 µM MLA, or 40 µM MLA for 8 h. The wound area was determined by capturing images through a microscope, and the variation in the wound area was measured using Image J software. Wound-healing ability of the cells was calculated using the following formula: (wound area at 0 h − wound area at 8 h)/wound area at 0 h.

### Transwell invasion assay

Cells were seeded in a 24-well insert-based culture (BD Biosciences) and were pre-coated with Matrigel Basement Membrane Matrix (BD Biosciences) 30 min prior to insertion in the well. After 24 h, the insert was removed and the membrane was cut. The number of invasive cells was counted using Image J software.

### Short hairpin RNA

A short hairpin RNA (shRNA) α7-nAChR and SNCG was purchased from the RNAi Core Facility of Academia Sinica (Taipei, Taiwan). The sh-α7-nAChR sequence was CCG GGC AAA TGT CTT GGA CAG ATC ACT CGA GTG ATC TGT CCA AGA CAT TTG CTT TTT TG, the sh-SNCG sequence was CCG GGA CCA AGG AGA ATG TTG TAC ACT CGA GTG TAC AAC ATT CTC CTT GGT CTT TTT TG and the negative control was Plko-1. Transfection was completed using Polyjet™ In Vitro DNA Transfection Reagent (SignaGen Laboratories, Medical Center Dr, Rockville, MD, USA) in accordance with the protocol provided.

### In vivo analysis of mouse xenografts

BALB/cAnN.Cg-Foxn1nu/Cr1Nar1 nude mice, male, aged 4–5 weeks and with an average body weight of 20 g, were purchased from the National Laboratory Animal Center, Taipei, Taiwan. All experiments on mice were performed according to the guidelines of our institute (Guide for Care and Use of Laboratory Animals, National Defense Medical Center) and were approved by the Institutional Animal Care and Use Committee of National Defense Medical Center, Taiwan (Approval No.: IACUC-15-365). Animal care and practice was based in the laboratory animal center of the National Defense Medical Center (Taipei, Taiwan). The 1 × 10^6^ OEC-M1 cells mixed with Matrigel (BD Biosciences)/phosphate-buffered saline (PBS) were injected subcutaneously into the flanks of the mice. The mice in each group were treated with 1.5 mg/kg/day [18], and PBS was used as the control. Tumor volume was calculated using the following formula: (length × width^2^)/2 [6].

### Immunohistochemistry

Tumor xenografts were extracted from formalin-fixed, paraffin-embedded tissue samples and were deparaffinized, and protein expression in each tissue was observed using the commercial Novolink™ polymer detection system (Leica Biosystems, Wetzlar, Hessen, Germany).

### Statistical analysis

GraphPad Prism (version 5) is used to perform statistical analyses and a P value < 0.05 is considered statistically significant. Data are expressed as the mean ± SD. The differences between groups are analyzed using a two-tailed student’s t-test when only two groups are present. Multiple groups will be analyzed with ANOVA.

## Results

### Nicotine up-regulates SNCG expression in OSCC cells

**Fig. 1:**
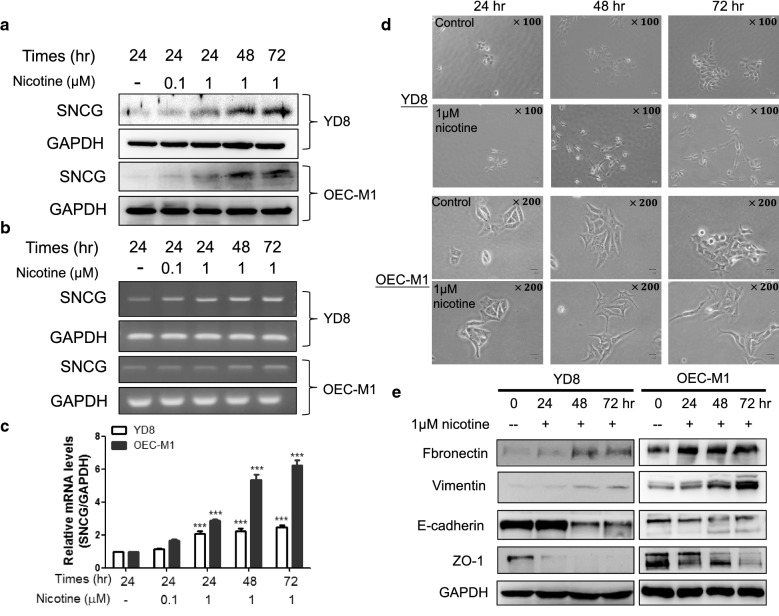
Nicotine induced SNCG expression and EMT change in OSCC cells. **a** Time- and dose-dependent increase SNCG protein expression in nicotine treated YD8 and OEC-M1 cells. Nicotine treatment also up-regulated SNCG mRNA expression in YD8 and OEC-M1 cells detected by **b** RT-PCR and **c** Q-PCR. **d** The morphology of control and nicotine-treated YD8 and OEC-M1 cells was investigated after 24, 48, and 72 h under microscope. Nicotine exposed YD8 and OEC-M1 cells displayed spindle morphology. **e** Western blot analysis of mesenchymal markers expression, such as fibronectin and vimentin was significant increase in YD8 cells and OEC-M1 cells with the time-dependent manner, whereas expression of the epithelial markers, ZO-1 and E-cadherin was decreased in both nicotine-treated YD8 and OEC-M1 cells. Data represent mean ± SD of 3 independent experiments. Statistical analysis was performed with ANOVA. *P < 0.05, **P < 0.01

To determine whether SNCG is a nicotine-responsive protein, YD8 and OEC-M1 OSCC cells were treated with different doses of nicotine for 72 h followed by Western blot analysis of SNCG levels. Treatment with nicotine stimulated SNCG expression in a dose-dependent manner (Fig. 1a). OSCC cells were also treated with nicotine for different time periods and it was observed that nicotine time-dependently upregulated SNCG expression (Fig. 1a). In addition, we investigated whether nicotine induces SNCG transcription. RT-PCR and quantitative RT-PCR demonstrated that nicotine significantly induced SNCG transcription (Fig. 1b, c). We also observed that nicotine treatment altered the morphology of oral cancer cells from an oval shape to a slender spindle shape (Fig. 1d), which is characteristic of epithelial–mesenchymal transition (EMT). Therefore, we further examined EMT markers. The results showed a time-dependent increase in epithelial markers such as E-cadherin and zonula occludens-1 (ZO-1) and a time-dependent decrease in mesenchymal markers such as fibronectin and vimentin in nicotine-treated OSCC cells (Fig. 1e).

### α7-nAChRs knockdown/inhibition suppresses SNCG expression

**Fig. 2:**
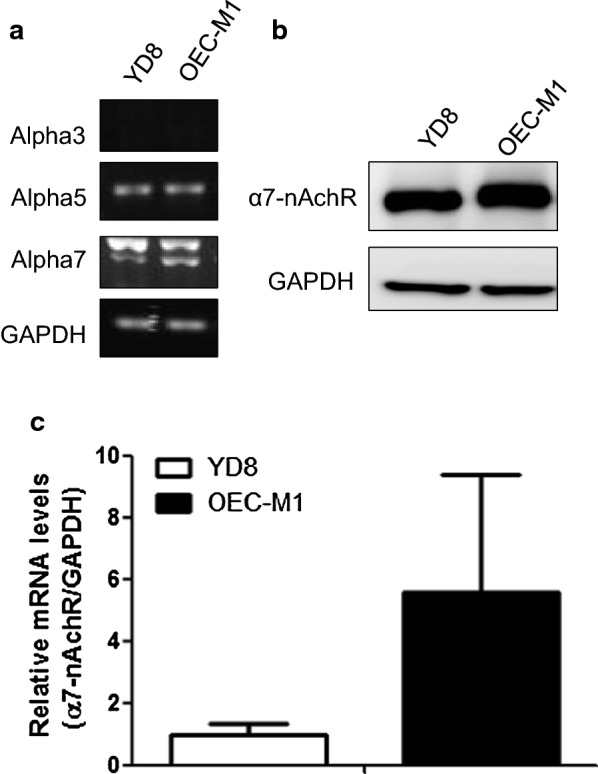
α7-nAChR expression level was abundant in YD8 and OEC-M1 cells. **a** The mRNA expression of the nAChR subunits in YD8 and OEC-M1 oral cancer cells. The α7-nAChR expression level was measured by **b** western blot, **c** PCR analysis in YD8 and OEC-M1 cells. Data represent mean ± SD of 3 independent experiments

**Fig. 3:**
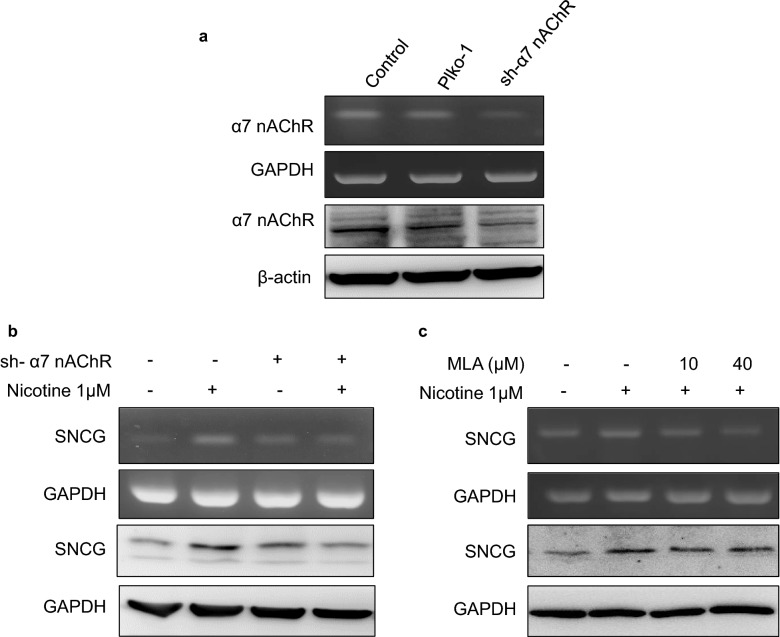
α7-nAChR knockdown/inhibition suppresses SNCG expression. **a** OEC-M1 cells were transfected with plko-1(vector only) or shRNA to α7-nAChR (sh α7-nAChR). Total RNA and proteins were harvested and subjected to RT-PCR and Western blot analysis of α7-nAChR expression. **b** OEC-M1 OSCC cancer cells were plated in cell culture dishes and treated with or without nicotine for 24 h. The cell lysates were harvested and subjected to RT-PCR and Western blot analysis of SNCG expression. GAPDH was also detected as loading control. **c** OEC-M1 cells were pretreated with or without MLA, an α7-nAChR inhibitor (MLA), followed by treatment with or without 1 µM nicotine for 24 h. Total RNA and proteins were harvested and subjected to RT-PCR and Western blot analysis of SNCG

In oral cancer, α3-, α5-, and α7-nAChRs have been identified as major receptors that mediate the effect of nicotine on tumor development [11, 19]. Therefore, we screened for these receptors in our tested cells and found that α3-nAChRs were not expressed in either cell line; moreover, both cell lines expressed similar levels of α5-nAChR not specific to any cell (Fig. 2a). α7-nAChR expression was abundant in YD8 and OEC-M1 cells (Fig. 2b, c). Therefore, we investigated the potential role of α7-nAChRs in mediating nicotine-induced upregulation of SNCG expression. We used short hairpin RNA (shRNA) to knockdown the expression of α7-nAChRs in OEC-M1 cells (Fig. 3a). Compared with control cells, the effect of nicotine on SNCG expression was attenuated in α7-nAChR-silenced cells (Fig. 3b). Similar results were observed in OEC-M1 cells treated with the α7-nAChR antagonist MLA (Fig. 3c).

### The AKT pathway is involved in nicotine-induced SNCG expression by α7-nAChRs

**Fig. 4:**
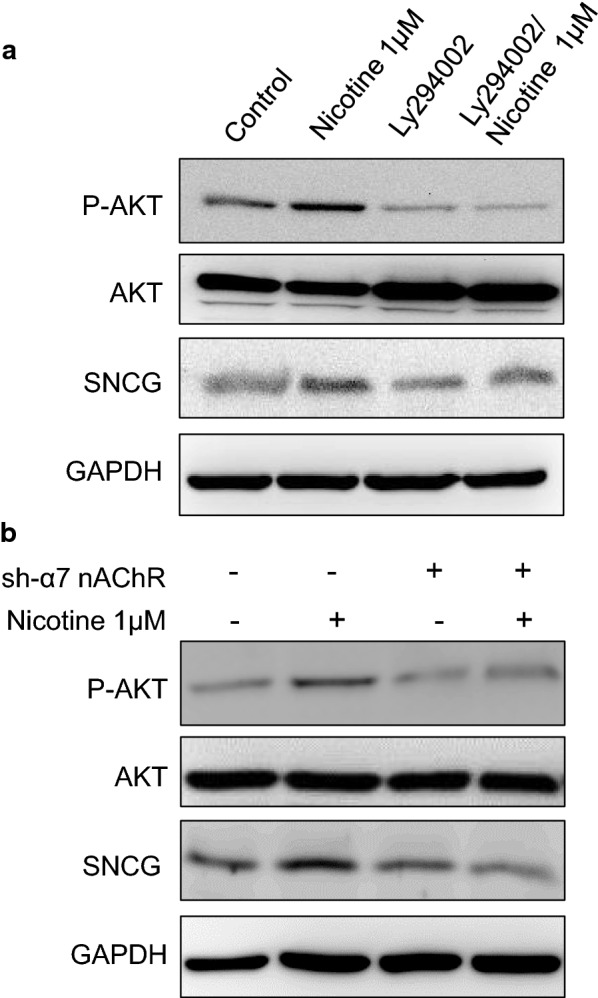
The α7-nAChR/AKT pathway is involved in nicotine-induced SNCG expression. **a** OEC-M1 cells were pretreated with or without 40 μM PI3K/AKT inhibitor then treated with or without 1 μM nicotine for 24 h. Total proteins were harvested and subjected to Western blot analysis of P-AKT, total AKT, and SNCG expression. GAPDH was also detected as loading control. **b** OEC-M1 cells with or without α7 nAChR knockdown were plated in cell culture dishes and treated with or without 1 μM nicotine for 24 h. The cell lysates were harvested and subjected to Western blot analysis of P-AKT, total AKT, and SNCG expression

In tumor progression the downstream signaling pathways of α7-nAChRs involve extracellular signal-regulated kinase (ERK) and AKT pathways [8, 20–22]. Therefore, in the current study, we examined whether exposure to nicotine causes activation of the AKT and/or ERK signaling pathways in OSCC cells. Notably, phospho-ERK levels did not change in nicotine-treated OSCC cells (data not shown). However, nicotine treatment increased phospho-AKT levels, and the effect of nicotine on SNCG expression was suppressed by treatment with ly294002 (Fig. 4a). Additionally, when α7-nAChRs were silenced, the effect of nicotine on AKT activation was suppressed (Fig. 4b). SNCG expression that was upregulated by nicotine treatment was attenuated when α7-nAChR was silenced (Fig. 4b). These results further confirm that nicotine increases SNCG expression by activating phospho-AKT through α7-nAChRs.

### Nicotine-induced tumor malignant behaviors were attenuated in SNCG knockdown cells

**Fig. 5:**
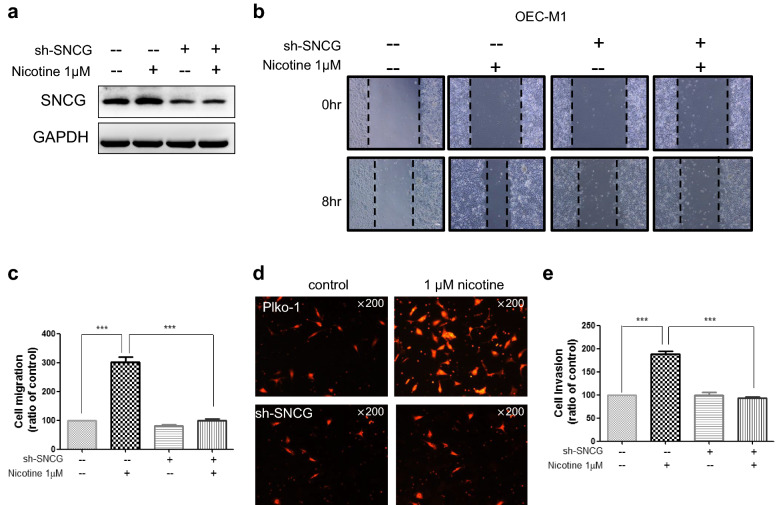
SNCG knockdown decrease nicotine-induced tumor malignant behaviors of OSCC. **a** shRNA efficiently silenced SNCG expression suppressed nicotine-induced SNCG expression. **b** Knockdown of SNCG suppressed nicotine-induced cell migration. **c** Quantitative results of wound-healing assay. **d** Nicotine induced cell invasiveness was inhibited in SNCG knockdown cells. **e** Quantitative results transwell invasion assay. Data represent mean ± SD of 3 independent experiments. Statistical analysis was performed with ANOVA. *P < 0.05, **P < 0.01, ***P < 0.001

To determine whether SNCG is involved in nicotine-induced cell migration and invasion, OSCC cells were treated with or without nicotine and transfected with SNCG shRNA (Fig. 5a), and then wound healing assays were performed. 8 h after treatment with 1 µM nicotine the wound area was much larger in vehicle-treated (control) cells compared with nicotine-treated cells. Cell migration was significantly increased by more than twofold in OEC-M1 cells, indicating that nicotine promoted OSCC cell migration (Fig. 5b, c). Knockdown of SNCG inhibited cell migration and abrogated the promotion of cell migration by nicotine (Fig. 5b, c). In addition, nicotine treatment increased the invasion ability of OEC-M1 cells threefold (Fig. 5d, e). Knockdown of SNCG abrogated the promotion of cell invasion by nicotine (Fig. 5d, e). This data demonstrates that SNCG is a mediator of nicotine-induced cell migration and invasion.

### Nicotine induces tumor growth and upregulation of SNCG in vivo

**Fig. 6:**
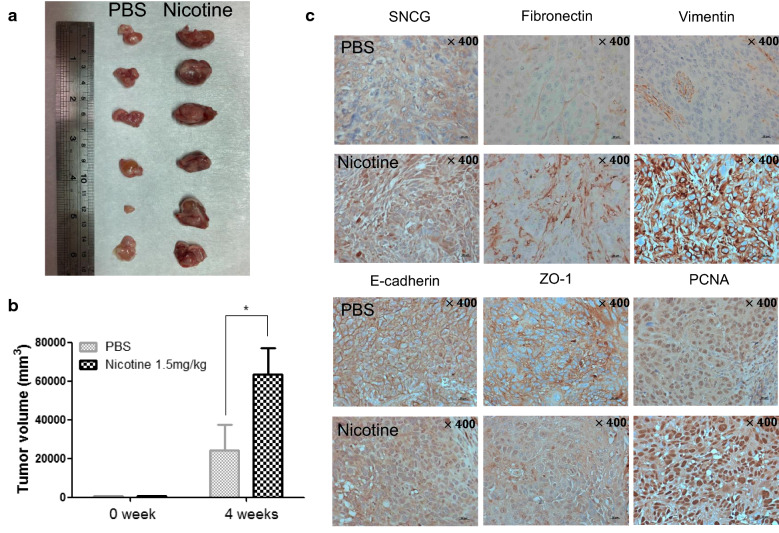
Nicotine upregulated SNCG expression and induced tumor growth in vivo. **a** Photograph of xenograft tumors. **b** Tumor volume was calculated by (length × width^2^)/2 mm^3^. **c** Immunohistochemistry of SNCG, PCNA, and EMT markers expression in nicotine and control groups. Statistical analysis was performed with ANOVA. *P < 0.05, **P < 0.01, ***P < 0.001. Original magnification: ×400 in (**c**)

Next, we used a xenograft model to determine whether nicotine induces SNCG expression and cancer malignancy. OSCC cells were subcutaneously injected into the flanks of nude mice treated with 1.5 mg/kg/day nicotine or PBS (control). After 4 weeks of tumor growth these mice were sacrificed. As displayed in Fig. 6a, nicotine significantly induced the growth of xenograft tumors in compared with the PBS group. Quantitative results of tumor volume are shown in Fig. 6b. Immunohistochemical analysis revealed increases in the immunoactivity of SNCG, fibronectin, vimentin and PCNA, and decreases in the immunoactivity of E-cadherin and ZO-1 in nicotine-treated nude mice (Fig. 6c). These findings indicate that nicotine-induced SNCG expression is strongly correlated with accelerated tumor proliferation and malignant effects in vivo.

## Discussion

In the present study, we reported that SNCG is a nicotine-responsive protein, and that nicotine binds to α7-nAChRs to activate PI3/AKT signaling, thereby upregulating SNCG expression. To the best of our knowledge, this study is the first to demonstrate that SNCG plays a crucial role in mediating nicotine-induced oral cancer malignancy. Our results demonstrate that SNCG is involved in nicotine-induced oral cancer malignancy. Nicotine induced time- and dose-dependent upregulation of SNCG expression in OSCC cells, and this effect was suppressed when α7-nAChR was knocked down or inhibited. Additionally, an in vivo xenograft model further showed that nicotine treatment augmented α7-nAChR and SNCG expression and tumor growth. Therefore, this study provides the biological mechanism underlying the association between nicotine and SNCG in oral cancer malignancy.

SNCG belongs to a family of small proteins called synucleins [12]. Synucleins are highly expressed in neuronal cells and have been specifically implicated in neurodegenerative diseases [23]. However, SNCG is clearly not involved in neurodegenerative diseases but is primarily involved in neoplastic diseases [12]. So far, the abnormal overexpression of the SNCG protein has been demonstrated in many different malignant diseases [2, 24–28]. For example, our previous study [2] showed a significant increase in SNCG expression in the OSCC cell lines and oral cancer tissues compared with the almost-undetectable expression levels in the nonneoplastic adjacent tissue. In agreement with previous reports on other tumors, it was found that SNCG expression increases from baseline levels in normal tissues to high levels in dysplasia and tumor tissues [26, 28, 29]. The present study further demonstrated that SNCG is a potential mediator of nicotine-induced malignant behaviors, such as invasion and migration. We analyzed retrieval in the GEO database, for adenocarcinoma of the lung tissue with or without cigarette smoking (GSE10072_209877); SNCG expression was higher in smoker’s tumor tissues compared with the tumors of those who had never smoked. Nicotine and its metabolites can promote tumor growth through increased proliferation, angiogenesis, migration, invasion and EMT. Many of those cancers are attributable to cigarette smoking and nicotine exposure. Notably, a recent study also reported that cigarette smoke extract (CSE) has strong inducing effects on SNCG gene expression in A549 lung cancer cells, and that CSE treatment augments the invasive capacity of A549 cells in an SNCG-dependent manner [30]. Our study reported that SNCG is involved in nicotine-induced oral cancer malignancy. Based on the aforementioned findings and our results, it can be hypothesized that SNCG also participates in other nicotine-related malignancies in patients who smoke. Therefore, additional studies should be conducted to investigate this hypothesis.

The present study demonstrated that SNCG participated in nicotine-induced OSCC malignancies and that knockdown of SNCG abolished nicotine-induced OEC-M1 invasion and migration. Our results demonstrate the oncogenic potential of SNCG in OSCC cells and support the notion that SNCG promotes cancer progression [2]. Although the precise mechanisms through which SNCG is involved in nicotine-induced oral cancer invasion and migration remain unclear, a recent report by Lee et al. revealed the biological function of SNCG in cancer. Lee et al. [31] reported that nicotine treatment upregulated matrix metalloproteinase (MMP) expression in cells and led to the degradation of the extracellular matrix (ECM). The ECM plays an important role in the malignant behaviors of cancer cells, including invasion and migration [32]. This role is achieved by the action of soluble, secreted proteases such as MMPs. SNCG has been shown to strongly induce MMP-9 and tissue inhibition of MMP-1 and MMP-2 (TIMP1 and TIMP2, respectively) expression and to moderately increase MMP-2 activity and protein levels [33]. Thus, nicotine-induced oral cancer malignancy may be caused by the upregulation of SNCG expression, and may lead to ECM degradation, which contributes to malignant behaviors, such as invasion and migration. In addition, an increasing number of studies have reported that endoplasmic reticulum (ER) stress and unfolded protein response (UPR) are activated in tumor cells and play critical roles in solid tumor growth and progression [34, 35]. The ER quality control machinery ensures the quality of proteins synthesized in the ER, so that only proteins which are properly folded and packaged into vesicles are destined for secretion, while those that are misfolded and do not pass the quality control checkpoints are delivered to the proteasome for degradation. Increase in the physiological demand for protein folding or stimuli that disrupt the ability of proteins to fold, cause the accumulation of un-/misfolded proteins in the ER lumen, resulting in ER stress [34, 35]. Recent reports have indicated that nicotine and SNCG are involved in ER stress induction and UPR [36, 37]. For example, a previous study [31] showed that nicotine treatment increased the levels of glucose-regulated protein-78 (GRP78; an ER stress indicator) and MMP (MMP-1, MMP-2, MMP-8, and MMP-9) expression. Hua et al. [38] reported that ER stress upregulated SNCG expression; this upregulation was mediated by activation transcription factor 4, an ER stress effector. Moreover, recent studies have demonstrated that SNCG acts as a chaperone protein and associates with BubR1 to over-ride mitotic arrest [39], and that SNCG enhances the ligand-binding affinity of estrogen receptors to activate mitotic signaling [40]. Because SNCG possesses chaperone-like activity [12], it may interact with different proteins in different cellular backgrounds. Identifying specific cellular targets of SNCG to determine the molecular mechanisms through which SNCG induces malignant behavior such as invasion and migration will provide insights into its oncogenic functions in OSCC.

## Conclusions

**Fig. 7:**
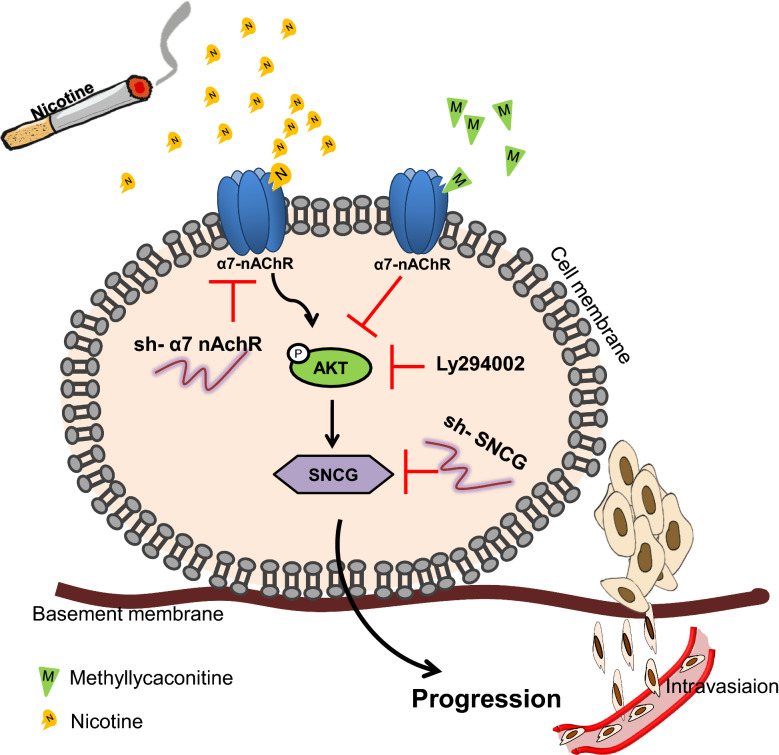
The summary model is depicted by which nicotine binds to α7-nAChRs to activate PI3/AKT signaling, thereby upregulating SNCG expression, and this effect was suppressed when α7-nAChR was knocked down or inhibited

Our findings revealed that nicotine and α7-nAChRs/PI3/AKT signaling positively regulated SNCG expression, which is an important signaling pathway that leads to the overexpression of the SNCG gene (Fig. 7). Our results indicated that SNCG participated in nicotine-induced malignant behaviors in OSCC cells, which were reversed by knockdown of SNCG. Therefore, blocking SNCG may be a beneficial strategy for treating patients with oral cancer who smoke.

## Data Availability

All data generated or analysed during this study are included in this published article.

## Abbreviations

Akt: Protein kinase B
DMSO: Dimethyl sulfoxide
EMT: Epithelial-to-mesenchymal transition
GAPDH: Glyceraldehyde 3-phosphate dehydrogenase
MLA: Methyllycaconitine
MTT: (3-(4,5-dimethylthiazol-2-yl)-2, 5-diphenyltetrazolium bromide)
nAChRs: Nicotinic acetylcholine receptors
OSCC: Oral squamous cell carcinoma
PBS: Phosphate-buffered saline
PCNA: Proliferating cell nuclear antigen
PCR: Polymerase chain reaction
PI3K: Phosphatidylinositol 3-kinase
QPCR: Quantitative Real-time PCR
sh-RNA: Short hairpin RNA
SNCG: γ-synuclein
Zo-1: Zonula occludens-1
