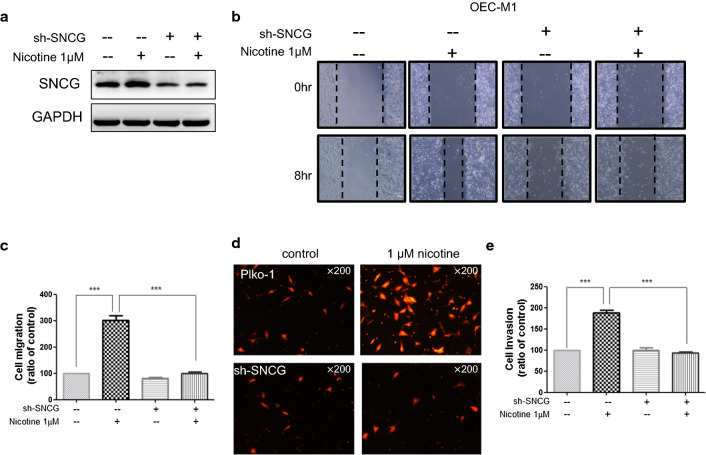# Correction to: Gamma synuclein is a novel nicotine responsive protein in oral cancer malignancy

**DOI:** 10.1186/s12935-020-01478-3

**Published:** 2020-08-26

**Authors:** Chia-Chen Hsu, Yu-Fu Su, Kuo-Yang Tsai, Feng-Chih Kuo, Chi-Fu Chiang, Chu-Yen Chien, Ying-Chen Chen, Chien-Hsing Lee, Yu-Chiao Wu, Kun Wang, Shyun-Yeu Liu, Yi-Shing Shieh

**Affiliations:** 1 Graduate Institute of Medical Sciences, National Defense Medical Center, Taipei, 114 Taiwan; 2 Department of Radiation Oncology, Tri-Service General Hospital, National Defense Medical Center, Taipei, 114 Taiwan; 3 Department of Oral and Maxillofacial Surgery, Changhua Christian Hospital, Changhua, 500 Taiwan; 4 College of Nursing and Health Science, Da-Yeh University, Chang-hua, 515 Taiwan; 5 Division of Endocrinology and Metabolism, Department of Internal Medicine, Tri-Service General Hospital, National Defense Medical Center, Taipei, 114 Taiwan; 6 Department of Dentistry, Tri-Service General Hospital, National Defense Medical Center, No. 161, Sec. 6, Min‑Chuan East Rd., Nei‑Hu, Taipei, 114 Taiwan; 7 Molecular and Cell Biology, Taiwan International Graduate Program, Academia Sinica and Graduate Institute of Life Science, National Defense Medical Center, Taipei, 114 Taiwan; 8 Department of Internal Medicine, Cardinal Tien Hospital and School of Medicine, College of Medicine, Fu Jen Catholic University, New Taipei City, Taiwan; 9 Department of Oral and Maxillofacial Surgery, Chi Mei Medical Center, Tainan, 710 Taiwan; 10 Department and Graduate Institute of Biochemistry, National Defense Medical Center, Taipei, 114 Taiwan

## Correction to: Cancer Cell Int (2020) 20:300 10.1186/s12935-020-01401-w

Following publication of the original article [1] the authors have notified us of an error in Fig. 5. The incorrect vs. correct Fig. 5 are presented below: 

Original Figure:
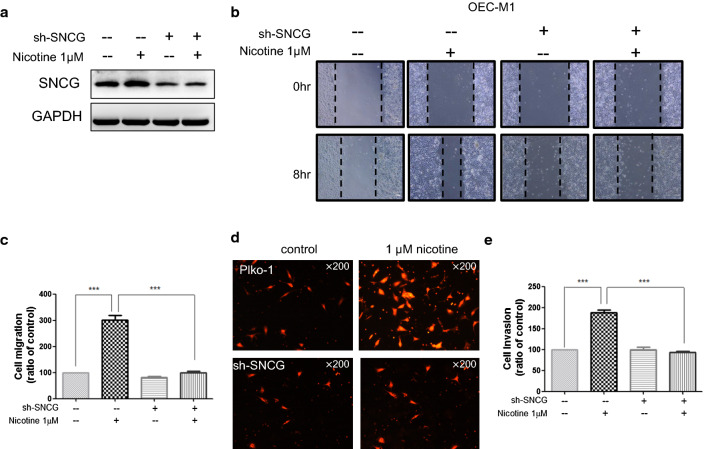


Correct Figure: